# Diagnostic accuracy and clinical applicability of the Swedish version of the 4AT assessment test for delirium detection, in a mixed patient population and setting

**DOI:** 10.1186/s12877-021-02493-3

**Published:** 2021-10-18

**Authors:** Yvonne A. Johansson, Theofanis Tsevis, Salmir Nasic, Catharina Gillsjö, Linda Johansson, Nenad Bogdanovic, Elisabeth Kenne Sarenmalm

**Affiliations:** 1 Skaraborg Hospital, Skövde, Sweden; 2 The Research School of Health and Welfare, Aging Research Network-Jönköping (ARN-J), Jönköping University, Jönköping, Sweden; 3 Theme Inflammation and Aging, Karolinska University Hospital, Stockholm, Sweden; 4 Department of Neurobiology, Care Sciences and Society, Karolinska Institutet, Stockholm, Sweden; 5 Department of Molecular and Clinical Medicine, Institute of Medicine, Sahlgrenska Academy, University of Gothenburg, Gothenburg, Sweden; 6 School of Health Sciences, University of Skövde, Skövde, Sweden; 7 College of Nursing, University of Rhode Island, Kingston, RI USA; 8 Institute of Gerontology, Aging Research Network-Jönköping (ARN-J), School of Health and Welfare, Jönköping University, Jönköping, Sweden; 9 Institute of Health and Care Science, Sahlgrenska Academy, Centre for Person-Centred Care Sahlgrenska Academy, University of Gothenburg, Gothenburg, Sweden

**Keywords:** Applicability, 4AT, Delirium, Diagnostic accuracy, Qualitative content analysis, Validation study

## Abstract

**Background:**

Delirium is common in older hospitalized patients. It has serious consequences e.g., poor health outcomes, mortality and increased costs. Despite that, many cases are undetected. Early detection of delirium is important in improving outcomes and use of assessment tools improves detection rates. The 4AT is a brief screening tool for delirium detection, which has not previously been translated into Swedish. The study aim was to evaluate diagnostic accuracy and clinical applicability of a Swedish version of the screening tool 4AT for delirium detection.

**Method:**

This diagnostic test accuracy study used a quantitative and a qualitative approach and evaluated the patients’ and the health care professionals’ experiences of the tool. Study included 200 patients ≥65 years from a university hospital and a county hospital in two Swedish regions. Medical specialties were geriatric stroke/neurology, geriatric multimorbidity, severe cognitive impairment, orthopaedic, and urology. The translated 4AT was tested against the reference standard DSM-IV-TR criteria, based on the Organic Brain Syndrome scale and patient records. The 4AT was assessed simultaneously and independently by two assessors. Additionally, data was collected through patient record reviews, and questions about applicability to the patients (*n* = 200) and the assessors (*n* = 37). Statistical analyses, and qualitative content analyses were conducted.

**Results:**

By reference standard 18% had delirium, and by 4AT 19%. The overall percent agreement was 88%, AUROC 0.808, sensitivity 0.70 (95% CI 0.51–0.84) and specificity 0.92 (95% CI 0.87–0.96). In the ward for severe cognitive impairment (*n* = 63) the 4AT was less sensitive and less specific. In the other wards (*n* = 132) sensitivity was 0.77 (95% CI 0.50–0.93), specificity 0.93 (95% CI 0.87–0.97), and AUROC 0.848. Interrater reliability (Kappa) was 0.918, *p* = < 0.001 (*n* = 144). The 4AT was well tolerated by patients, easy to use for health care professionals, and took a few minutes to conduct.

**Conclusion:**

The Swedish version of 4AT is an accurate and applicable tool to use in clinical practice for detecting delirium in hospitalized patients across different medical specialities, and to use by different professionals and levels of seniority. To improve patient outcomes, we recommend the 4AT to be incorporated in clinical practice in health care settings in Sweden.

## Background

Delirium is a common acute and severe neuropsychiatric disorder associated with a variety of adverse outcomes [1]. Despite this, delirium is often underdiagnosed [1–6], poorly understood and managed [1, 2, 5, 7], especially in the most vulnerable and frail [5]. Adverse outcomes caused by delirium include stressful experience, emotional suffering and distress [8], complication of medical conditions, prolonged hospital stays, increased mortality [5], increased healthcare costs [9], and a great risk of developing dementia, especially in the oldest-old [10].

The highest incidence and prevalence of delirium occur among older hospitalized patients and vary according to patient group and type of care. In a meta-analysis of 33 studies an overall prevalence of 23% was found in older hospitalized patients [11]. Delirium and cognitive impairment, e.g., dementia, are strongly intertwined. Cognitive impairment is the strongest risk factor for delirium and delirium may trigger or worsen cognitive impairment and preexisting dementia [5, 12, 13]. Additionally, one sign that manifests in delirium is cognitive impairment, and delirium is often mistaken for dementia although dementia undergoes a progressive deterioration process [13]. Subtypes of delirium are hyperactive, hypoactive, and mixed delirium [14]. In most cases, delirium is triggered by treatable underlying causes, e.g., acute illness [5, 14]. It is essential to prevent delirium incidence due to its common occurrence and association to poor outcomes [15]. The incidence might be reduced in 30% [16] to 40% by providing good fundamental care, such as early mobilization and promotion of sleep [2].

Delirium is a clinical bedside diagnosis, and knowledge about the signs and symptoms of delirium, as well as rapid detection of delirium, are required to prevent poor outcomes [4, 17]. Despite this, about 70% of all cases of delirium are unidentified in acute care hospitals [3, 5, 18, 19]. This is due to a lack of consensus, awareness and knowledge of delirium, as well as negative attitudes [20] and the use of mainly subjective assessments with poor diagnostic accuracy [3, 6, 7, 21]. To facilitate the process of identifying delirium, the use of a brief bedside screening tool is reasonable as a first stage, with a more detailed diagnosis assessment of patients identified in the first stage [22]. An internationally commonly used assessment tool for delirium is the Confusion Assessment Method (CAM) [23]. To use the tool correctly, regular training and separate testing of cognitive function are required [23, 24], which makes the CAM less feasible for routine use in acute care settings [25, 26]. Three assessment tools for delirium are available in Swedish, two of them developed in Sweden: the Organic Brain Syndrome scale (OBS scale) [27, 28] and the Confusional State Evaluation (CSE) [29]. These tools are relatively extensive, and mainly used in research. The third instrument, the Swedish version of the Nursing Delirium Screening Scale (NuDesc) [30], has shown low sensitivity (47–66%). Moreover, regular training and separate cognitive testing are recommended [31].

A brief screening tool developed and designed for the detection of delirium in routine clinical practice is the 4AT (4 A’s test). The tool consists of the items Alertness, Abbreviated Mental Test-4 (AMT4), Attention, and Acute changes. The 4AT can be used by any healthcare professional at the first contact with the patient, and at any other time when delirium is suspected [32]. The tool is an episodic tool, which means it is not designed for daily monitoring multiple times per day or daily use for prolonged periods, because of patient burden and cognitive test practice effects [1]. Regular training is not required. Two existing brief tests for cognitive function, the AMT4 and the Months Backwards test, are incorporated in the 4AT to avoid separate cognitive testing [32]. The 4AT acts as a screening tool and does not provide a formal diagnosis. A score that indicates delirium should thus trigger a more detailed assessment by a suitably qualified professional [33]. In addition, the 4AT has been used as a single tool for delirium detection [34–36]. Several studies in different countries have shown that the 4AT is a sensitive (76–93%) and specific (70–94%) screening tool for delirium detection in geriatric inpatients [22, 32, 33, 37]. The 4AT has so far not been used in Sweden.

Although several assessment tools for delirium detection have been developed, few studies have evaluated the clinical applicability of using these tools, either from the perspective of patients or of healthcare professionals. Essential for the success of clinical interventions is further qualitative exploration of the use of diagnostic tools [38]. In Sweden, the lack of validated brief screening tools for delirium detection adapted to acute care hospital settings may hamper early detection of delirium and improvements in patient care and outcomes. Therefore, the aim of this study was to evaluate the diagnostic accuracy and clinical applicability of a Swedish version of the 4AT screening tool for delirium detection.

## Method

### Design

This was a diagnostic test accuracy study with both a quantitative and a qualitative approach. The study followed the Standards for Reporting of Diagnostic Accuracy Studies guidelines, the STARD, 2015 [39, 40].

### Setting and sample

The study was conducted at a university hospital and a county hospital. Specialist geriatric care is provided at the university hospital but not specifically in the county hospital. Patients were recruited consecutively from wards with an expected high occurrence of patients with delirium; in the university hospital from one geriatric clinic including three non-surgical wards: geriatric stroke/neurology*, geriatric multi-morbidity*, and severe cognitive impairment, and in the county hospital from two surgical wards: orthopedics* and urology* (*in this study called general wards). The patients in the geriatric wards for stroke/neurology and multimorbidity are transferred from other wards because of the need of additional geriatric care. The ward for severe cognitive impairment is a specialist ward for patients with moderate or severe dementia, or severe delirium where the patients are enrolled directly from the ED, from other wards or electively. In the wards in the county hospital, the patients are acutely as well as electively admitted.

The sample size was calculated to evaluate the concordance between the 4AT and the reference standard. Assuming that the area under the receiver operating characteristic curve (AUROC) was 0.75 or higher (cutoff ≥4 for 4AT) and that the occurrence of delirium was 20% [14] in the county hospital, 65 patients were needed for 80% power and a 5% significance level. For a significance level of 1%, 95 patients were required. To compensate for potential dropouts, e.g., indeterminate diagnoses and subgroup analyses, 100 patients per hospital were included (*n* = 200). The inclusion criteria were patients aged ≥65 years with admission to the selected hospital wards the day before or on the same day as the assessment, or within 24 h post-surgery. The exclusion criteria were significant hearing or visual impairment, terminal illness, coma, aphasia, and inability to understand and answer questions in Swedish. In addition, the assessors (*n* = 37) that conducted the assessments with the 4AT were included in the applicability part of the study.

### Measurements

#### Demographic and clinical data

The demographic and clinical data of the patients included age, gender, admission type, length of hospital stay, main diagnosis, dementia diagnosis, and coexisting medical conditions, according to the International Statistical Classification of Diseases and Related Health Problems - Tenth Revision (ICD-10) [41].

The demographic data of the assessors included gender, profession, and the number of years of professional experience and years at the current workplace.

#### Diagnostic accuracy

##### Index test

*The 4AT*: The 4AT contains four items (subscales). The first item assesses alertness by observation (0; 4). Items 2–3 are existing tests for cognitive function: the Abbreviated Mental Test-4 (AMT4), where the patients state their own age and date of birth, the present location, and the current year (0–2), and the Months Backwards test that assesses attention by asking the patient to name the months of the year in backward order (0–2) [25, 32, 42, 43]. Item 4 evaluates significant acute changes or a fluctuating course of alertness, cognition, or other mental function, for instance, paranoia and hallucinations developing over the past 2 weeks and still evident in the last 24 h (0; 4). For this, information from several sources may be required, e.g., from other professionals, next of kin, and patient records. The total score ranges from 0 to 12, where 0 indicates that delirium or severe cognitive impairment is unlikely; 1–3 indicates possible cognitive impairment; and ≥ 4 indicates possible delirium +/− cognitive impairment [32]. In this study, a score < 4 indicated unlikely delirium, and ≥ 4 indicated possible delirium, as defined by Shenkin et al. [33].

After obtaining approval from professor MacLullich Edinburgh University, the translation process of the 4AT followed a standard forward-backward translation procedure [44]. The 4AT (version 1.2) [45] was independently translated into Swedish by two of the authors (YJ, EKS) and two persons skilled in English, one assistant nurse experienced in delirium care and one research manager. The different versions were discussed by the four persons until consensus was reached. The Swedish version was translated back into English by an independent bilingual translator who had no knowledge of the original tool. There were a few discrepancies between the back-translated version and the original English version, and these discrepancies were discussed by the four initial translators. Two of the authors (TT, NB), specialists in neuro-geriatric medicine and experienced in delirium care, agreed on the final Swedish version.

##### Reference standard

The criteria for delirium in the Diagnostic and Statistical Manual on Mental Disorders, 4th ed. Text Revision (DSM-IV-TR) [46], was used as the reference standard based on information derived from the OBS scale [27, 28] and patient records.

*The OBS scale* is an interview and observation scale developed for clinical evaluation of various behavioral, psychiatric and emotional symptoms and signs appearing in organic brain disorders in older people, such as dementia or delirium [28, 47]. This reference standard procedure has previously been used in several Swedish studies, e.g., Björkman Björkelund, 2006 [28], Edlund et al., 2007 [48], Lingehall, 2017 [31], Smulter et al., 2019 [6]. The OBS scale (score 0–165) is divided into two parts. Part I, the disorientation subscale (score 0–48), reflects a short-time perspective and comprises 16 questions about the patient’s awareness of and orientation to own identity, time and place, and knowledge about some general topics. Part II, the confusion subscale (score 0–117), evaluates the last 7 days, covering a wide spectrum of psychopathology in 39 clinical items. The subscale reflects the severity and variation of the signs and symptoms of the clinical state, suspiciousness, emotional reactions, language and speech difficulties, delusions and hallucinations, neurological symptoms, spatial orientation and recognition, physical and practical ability, and social interaction skills. No cutoff is suggested. The items and their ratings are described in detail in e.g., Björkman Björkelund et al. [28].

*The DSM-IV-TR criteria* for the diagnosis of delirium are disturbance of consciousness (i.e., reduced clarity of awareness of the environment) with reduced ability to focus, sustain or shift attention, and disturbances in cognition that develop over a short period of time and represent an acute change that tends to fluctuate in severity. The disturbances are not better explained by another preexisting cognitive disorder. Evidence is required that the disturbance is a direct physiological consequence of another medical condition, substance intoxication or withdrawal, or is due to multiple etiologies [46].

#### Clinical applicability

The time duration of the assessments with the 4AT and the OBS scale was measured by the assessors in a subsample of 100 patients (county hospital).

Experiences of the 4AT were evaluated from the patients’ and the assessors’ perspective in the two hospitals. Open-ended questions were chosen as they allow people to respond in their own words [49]. The patients were asked the question “How did you experience answering the questions that were asked before?” The questionnaire to the assessors consisted of the assessors’ experiences of asking the questions in the 4AT, the patients’ reactions to answering the questions, using the 4AT compared with not using a screening tool, and the advantages and disadvantages of using the 4AT as a routine procedure in clinical practice.

### Data collection

After clinical consideration by Registered Nurses (RNs), patients were included or excluded on the assessment days. Patients who fulfilled the inclusion criteria received oral and written information that described the aim and content of the study. Informed oral consent was used as patients with cognitive impairment might experience discomfort when signing documents [50]. The consents were obtained prior to the administration of the 4AT assessment. In the cases where the patient was unable of giving consent, the patient’s representative was consulted about the consent. The oral consent was registered, as well as whom who obtained the consent. The assessments were conducted during daytime (09:00–16:00) to avoid influence of the time of day on cognitive function. Recruitment took place on selected assessment days (Monday - Friday) from May 14, 2018, and continued until 100 patients were included in each hospital, at the university hospital in February 8, 2019 and at the county hospital in September 4, 2018 (except for June 21–August 20, 2018). At the university hospital, the 4AT assessments were conducted by clinical RNs, clinical physicians, and two of the authors (TT, NB) (*n* = 34) at the same time as the 4AT was implemented in clinical practice, while research nurses (*n* = 3) conducted the assessments with the 4AT at the county hospital throughout the study.

Data were collected through structured patient interviews and observations based on the instruments used in the study, and from patient records. Additionally, the patients were asked about their experiences of answering the questions in the 4AT before the OBS scale assessment started. Finally, a questionnaire addressing the assessors’ (*n* = 37) experiences of using the 4AT was administered when data collection was completed, with one reminder.

Before the study started, the assessors were provided with a brief basic introduction to delirium and the 4AT, likewise new employees during the study period. The 4AT was assessed simultaneously and independently by two assessors, to avoid any bias due to the fluctuating nature of delirium. The target was to perform the assessment with the OBS scale as soon as possible after the assessment with the 4AT, preferably within 30 min, taking into account the patient’s capacity and the level of exhaustion experienced when responding to the questions about the demographic variables and the 4AT. The time between the assessments with the 4AT and the OBS scale measured in the county hospital (*n* = 100) was in median 13 min 30 s (QL-QU 9:00–24:45) with equal medium time for the patients with and without dementia. All assessments were blinded, and the assessors had no knowledge of the study test results. An overview of the informed consent and data collection in chronological order is shown in Table 1.

**Table 1:** Overview of informed consent and data collection in chronological order

Activity		Oral and written information	Informed oral consent	Demographic data, and 4AT	Question about 4AT, and theOBS scale	Clinical data from patient records	Questionnaire assessors
**Providers**	University Hospital	Researchers^a^,^b^, physicians or RN (*n* = 34)	Researchers^a^,^b^, physicians or RN (*n* = 34)	Researchers^a^,^b^, physicians or RN (n = 34)	Researcher^a^ or one research nurse^d^ (*n* = 2)	Researcher^a^ or one research nurse^d^ (*n* = 2)	Researcher^a^(*n* = 1)
	County Hospital	Nursing coordinator/manager. Relocated patients: Research nurses (n = 3)	Research nurses (n = 3)	Research Nurses(n = 3)	Researcher^c^ (n = 1)	Researcher^c^ (n = 1)	Researcher^c^ (n = 1)

Researcher ^a–c^: ^a^ = TT, ^b^ = NB, ^c^ = YJ. ^d^ = One research nurse specialized in delirium care

### Data analyses

Descriptive statistics were calculated for all data. For associations between numerical variables of the ordinal data type, Spearman’s correlation was used. Mann-Whitney U-tests were used for comparisons between the groups with respect to numerical variables, and the Chi-square test was used for comparisons with respect to categorical variables. In five patients, the assessments of delirium with the 4AT did not yield a distinct and unitary diagnosis. These data were handled as indeterminate and removed from the diagnostic accuracy analysis, except for the analysis of interrater reliability. A *p-*value of 0.05 (2-tailed) was considered statistically significant. The 95% confidence interval was reported for the estimates. All statistical analyses were carried out using the SPSS Statistics software, Version 25.0 for Windows (IBM Corp, Armonk, New York, USA).

#### Diagnostic accuracy

##### Comparison between the subscales in the 4AT and the OBS scale

Correlation between the scores in the four subscales in the 4AT and the scores in the OBS scale, and the items in the OBS scale part II, were calculated with Spearman’s correlation. As there is no cutoff in the OBS scale, the data were divided into two groups based on the dichotomized 4AT (4AT < 4 and ≥ 4) and the corresponding scores of the OBS scale (part I, part II and the total OBS scale). The differences between the two groups were illustrated in a boxplot and calculated using the Mann-Whitney U-test.

##### The index test accuracy versus the reference standard

After that all data had been collected in the study, the delirium diagnosis was determined retrospectively by four of the authors (YJ, TT, NB, EKS), two of whom are physicians with their specialization in neuro-geriatric medicine (TT, NB), and with long clinical experience of using the DSM-IV-TR criteria. Blinded to the results of the 4AT, the four authors independently evaluated the results on the OBS scale and the patient records to decide whether the patient met the DSM-IV-TR criteria for delirium. A definitive diagnosis of delirium was reached in consensus discussions.

In the comparison between the 4AT index test and the DSM-IV-TR reference standard, the original cutoff ≥4 for delirium in the 4AT was used. The AUROC was calculated, as well as the overall percentage agreement (OPA), the sensitivity, specificity, and the positive (PPV) and negative predictive values (NPV). To establish the overall performance of the 4AT, Youden’s Index (*J*) was calculated, where 0 = no value, and 1 = perfect test [51, 52]. These analyses were performed on the total sample and the participating hospitals and wards, and on the subgroups of patients with and without dementia.

##### Interrater reliability

The Kappa coefficient of the paired assessments of the 4AT was calculated. The Kappa can be calculated if ≥ two paired assessments are performed by two assessors, and if all categories in a test are represented by each assessor. Interrater reliability was calculated on the total sample as well as on the two hospitals respectively.

#### Clinical applicability

##### Time duration

Time durations for the assessments with the 4AT and the OBS scale in the subsample (*n* = 100) were calculated and reported as means. Differences between the time duration of the 4AT for patients with and without delirium or dementia were calculated using the Mann-Whitney U test.

##### Experiences of the 4AT

The patients’ experiences of being evaluated with the 4AT and the assessors’ response of using the 4AT were separately analyzed with manifest qualitative analysis according to Elo and Kyngäs [53]. This analysis included open coding, the creation of subcategories and generic categories based on similarities and differences in the content. Codes, subcategories and generic categories were continuously moved back and forth and checked against the original text. Finally, the number of patient and assessor experiences were counted per category and the data were presented separately. The categories are reported in the text, and quotes are used to further illuminate and validate the categories.

## Results

### Demographic and clinical characteristics

**Fig. 1:**
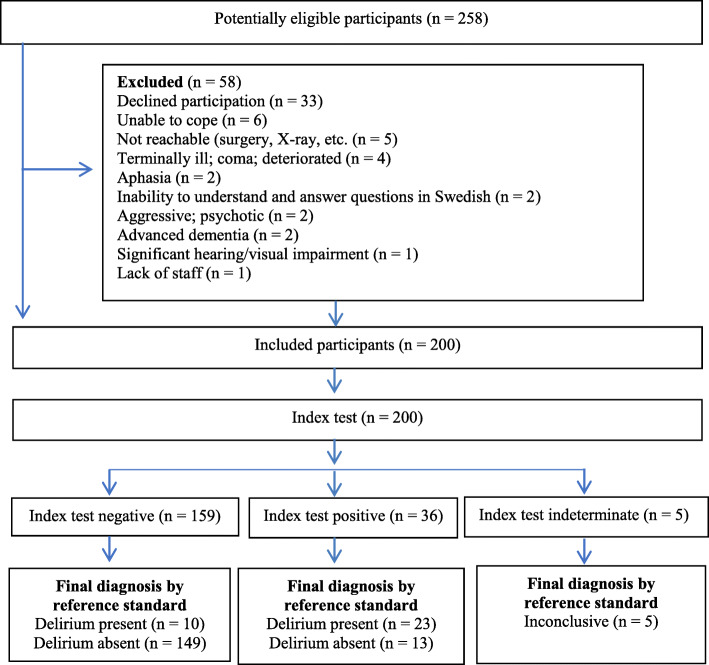
STARD flow diagram (total across the two hospitals, *n* = 200)

There were 258 potentially eligible patients on assessment days, 58 of whom were excluded (Fig. 1). In total, 200 patients were included in the study. Demographic and clinical characteristics are presented in Table 2. According to the reference standard, delirium was present in 36 of 200 patients (18, 95% CI = 13–24%), 21 at the university hospital and 15 at the county hospital. The highest prevalence of delirium occurred in the wards for severe cognitive impairment (28%) and orthopedics (19%). Patients with delirium were older, had a higher prevalence of dementia, and 2 days longer hospital stays than patients without delirium. According to the patient records, 52 (26%) patients had dementia (37 patients in the university hospital, 33 of whom were in the ward for severe cognitive impairment, and 15 patients in the county hospital). Of those with dementia, 15 (29%) patients were diagnosed with delirium according to the reference standard (eight in the university hospital and seven in the county hospital). In total, 73 (37%) patients had dementia, delirium or a combination of both. In addition, 16 (8%) patients displayed signs and symptoms of mild cognitive impairment according to their records. The most common main diagnoses were fracture related to fall (*n* = 86/43%), delirium (*n* = 29/15%), and dementia (*n* = 19/10%).

**Table 2:** Demographic and clinical characteristics for older inpatients, divided by delirium status (reference standard) (*n* = 200)

	Total (*n* = 200)	Delirium absent *n* = 164 (82%)	Delirium present *n* = 36 (18%)	*p* value
*Age*, median (QL-QU)^a^	80 (73–87)	79 (73–85)	85 (78–88)	0.019^d^
*Gender*, n (%)				0.439^e^
Men	105 (53%)	84 (51%)	21 (58%)	
Women	95 (48%)	80 (49%)	15 (42%)	
*Ward specialty*, n (%)				0.023^e^
Severe cognitive impairment	67 (34%)	48 (29%)	19 (53%)	
Geriatric stroke/neurology	17 (9%)	16 (10%)	1 (3%)	
Geriatric multi-morbidity	16 (8%)	15 (9%)	1 (3%)	
Orthopedics	67 (34%)	54 (33%)	13 (36%)	
Urology	33 (17%)	31 (19%)	2 (6%)	
*Admission to ward, n (%)*				0.436^e^
Acute^b^	157 (79%)	127 (77%)	30 (83%)	
Elective	43 (22%)	37 (23%)	6 (17%)	
*Length of hospital stay*, median (QL-QU)^a^	9 (6–13)	8 (5–13)	10 (7.5–15)	0.011^d^
*Dementia* at discharge^c^ n (%)	52 (26%)	37 (23%)	15 (42%)	0.018^e^

^a^ QL-QU = Quartile, Lower – Quartile, Upper

^b^ From Emergency Department (ED), other ward, other hospital or directly from own home

^c^ As main diagnosis or comorbidity, with ICD-10 code or reported in record text

^d^ Based on Mann-Whitney U-test (continuous variables)

^e^ Based on (Pearson) Chi-Square test (categorical variables)

In the 4AT index test, where all items were completed (*n* = 200), an indeterminate diagnosis occurred in five (3%) patients due to differences in the scoring of attention/month backwards (*n* = 4), and/or acute changes or fluctuating course (*n* = 3) in the paired assessments. These five patients were removed from the analyses of diagnostic accuracy, except for the analysis of interrater reliability. In the remaining 195 patients, 33 patients (17%, 95% CI = 12–23%), had delirium according to the DSM-IV-TR reference standard criteria (18/95 in the university hospital and 15/100 in the county hospital).

According to the 4AT index test, 36 of 195 patients (19%) had delirium. Of the patients with delirium in the 4AT (*n* = 36), 23 had delirium, according to the reference standard; thus, the 4AT falsely detected 13 non-delirious patients as delirious, according to the reference standard. According to patient records, these patients had dementia (*n* = 7), mild cognitive impairment (n = 4), or brain tumor (*n* = 1). Additionally, one patient had delirium as the main diagnosis. Moreover, the 4AT missed ten patients with delirium according to the reference standard. These were patients with dementia (n = 3), mild cognitive impairment (*n* = 5), Lyme disease (1), or delirium (n = 1), according to their records. Several of these 23 patients also had other diseases that may have affected the scoring, e.g., Parkinson’s disease, overconsumption of alcohol, and previous TIA and stroke. Delirium present, delirium absent or inconclusive are presented in the flow chart (Fig. 1).

Of the 37 assessors, 14 (38%) responded to how they experienced using the 4AT, 4 physicians and 10 RNs. Twelve assessors worked in the university hospital, 6 of whom in the general wards. The assessors had worked 1–38 years (mean 11.6) in their profession and 0.5–19 years (mean 8.7) in the current workplace.

### Diagnostic accuracy

#### Comparison of the subscales in the 4AT and the OBS scale

The analysis of correlations between the scores in the subscales in the 4AT and the subscales in the OBS scale showed that there were positive correlations between the 4AT and the OBS scale, except with regard to neurological symptoms in part II of the OBS scale. Alertness showed weak correlation with the OBS scale, but all patients except two were assessed as having normal alertness (score 0). The two incorporated tests for cognitive function had moderate to strong correlation with the disorientation subscale, but also moderate correlation with the confusion subscale and the items spatial orientation-recognition, social interaction skills, and language-speech difficulties. The item acute change showed the strongest correlation with the confusion subscale and the items delusions and hallucinations, emotional reactions, and spatial orientation-recognition (Table 3).

**Table 3:** Correlations (Spearman’s coefficient) between the subscales in the 4AT and the subscales and items in the OBS scale (*n* = 200)

Instruments	4AT subscales			
The OBS scale	Alertness	AMT-4	Months backwards	Acute change
OBS total score	0.168^a^	0.766^b^	0.629^b^	0.317^b^
OBS part I (disorientation subscale)	0.171^a^	0.771^b^	0.616^b^	0.241^a^
OBS part II (confusion subscale)	0.164^a^	0.614^b^	0.525^b^	0.360^b^
Clinical state	0.187^b^	0.267^b^	0.279^b^	0.254^b^
Suspiciousness	n.s.	0.312^b^	0.279^b^	n.s.
Emotional reactions	n.s.	0.375^b^	0.304^b^	0.292^b^
Language-speech difficulties	0.165^a^	0.577^b^	0.496^b^	0.239^b^
Delusions and hallucinations	n.s.	0.217^b^	0.192^b^	0.354^b^
Neurological symptoms	n.s.	n.s.	n.s.	n.s.
Spatial orientation-recognition	0.196^b^	0.654^b^	0.528^b^	0.273^b^
Social interaction skills	0.180^b^	0.465^b^	0.522^b^	0.265^b^

^a^significant at 0.05 level / ^b^significant at 0.01 level

**Fig. 2:**
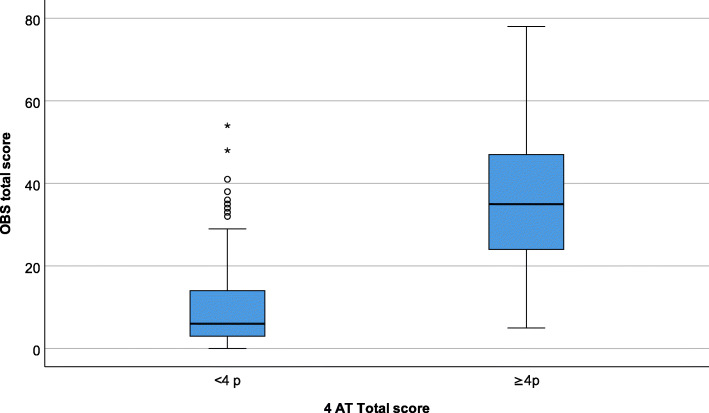
Boxplot distribution of the OBS scale total score according to the dichotomized 4AT (cutoff ≥4) (*n* = 200)

Boxplot distribution of the total score of the OBS scale in the dichotomized 4AT (cutoff ≥4) (*n* = 200) is shown in Fig. 2. The extreme values (*n* = 10) were seen in the wards for severe cognitive impairment (*n* = 7), orthopedics (*n* = 2), and geriatric stroke/neurology (*n* = 1). They consisted of patients with dementia (*n* = 4) or mild cognitive impairment (*n* = 2) as the main diagnosis or comorbidity, or delirium (*n* = 4) as the main diagnosis in their records. Seven of these ten patients also had other diseases that may have affected the scoring on the OBS scale, e.g., Parkinson’s disease, overconsumption of alcohol, memory problems, and previous TIA and stroke. The comparison between the dichotomized 4AT and the corresponding scores on the OBS scale, part I, part II and the total OBS scale (Mann-Whitney U test, *p =* < 0.001), is reported in Table 4.

**Table 4:** Comparison between the dichotomized 4AT and the corresponding scores on the OBS scale (*n* = 200)

	4AT < 4p (***n*** = 162)	4AT ≥ 4p (***n*** = 38)	***p*** value^c^
	Median (QL-QU^b^)	Median (QL-QU^b^)	
**OBS Total score**^**a**^	6 (3–14)	35 (24–48)	< 0.001
**OBS score part I**	3 (0–9)	24 (12–29)	< 0.001
**OBS score part II**	2 (0–5)	14 (8–20)	< 0.001

^a^ Total score = OBS scale part I + part II

^b^ QL-QU = Quartile, Lower – Quartile, Upper

^c^ Based on the Mann-Whitney U test

#### The index test accuracy versus the reference standard

With the cutoff ≥4, the AUROC for the total sample (*n* = 195) was 0.808 (95% CI = 0.746–0.861). All estimates were lower in the ward for severe cognitive impairment compared with the other wards, whereas the PPV was higher. In patients with dementia (*n* = 52), the 4AT was more sensitive and less specific. The Youden index for the total sample was 0.617, with a higher index in the general wards in both hospitals (0.695) (Table 5).

**Table 5:** Index test 4AT (cutoff ≥4) against reference standard DSM-IV-TR criteria (OBS scale and patient records), indeterminate diagnosis excluded, *n* = 195

Comparisons	True pos.	True neg.	False pos.	False neg.	OPA %	Sensitivity (95% CI)	Specificity(95% CI)	PPV(95% CI)	NPV(95% CI)	Youden’s Index	AUROC(95% CI)
Total sample (*n* = 195)	23	149	13	10	88%	0.70 (0.51–0.84)	0.92 (0.87–0.96)	0.64 (0.50–0.76)	0.94 (0.90–0.96)	0.617	0.808 (0.746–0.861)
University hospital (*n* = 95)	12	71	6	6	87%	0.67 (0.41–0.87)	0.92 (0.84–0.97)	0.67 (0.47–0.82)	0.92 (0.86–0.96)	0.589	0.794 (0.699–0.870)
County hospital (*n* = 100)	11	78	7	4	89%	0.73 (0.45–0.92)	0.92 (0.84–0.97)	0.61 (0.42–0.77)	0.95 (0.89–0.98)	0.651	0.825 (0.737–0.894)
University hospital, special ward^a^ (*n* = 63)	10	42	5	6	82%	0.63 (0.35–0.85)	0.89 (0.77–0.96)	0.67 (0.45–0.83)	0.88 (0.79–0.93)	0.519	0.759 (0.635–0.858)
University hospital, general wards^b^ (*n* = 32)	2	29	1	0	97%	1.00 (0.16–1.00)	0.97 (0.83–1.00)	0.67 (0.23–0.93)	1.00n.a.	0.967	0.983 (0.861–1.00)
Both hospitals, general wards^c^ (*n* = 132)	13	107	8	4	91%	0–77 (0.50–0.93)	0.93 (0.87–0.97)	0.62 (0.44–0.77)	0.96 (0.92–0.98)	0.695	0.848 (0.775–0.904)
Patients with dementia (*n* = 50)	11	30	6	3	82%	0.79 (0.49–0.95)	0.83 (0.67–0.94)	0.65 (0.46–0.80)	0.91 (0.78–0.97)	0.619	0.810 (0.674–0.907)
Patients without dementia (*n* = 145)	12	119	7	7	90%	0.63 (0.38–0.84)	0.94 (0.89–0.98)	0.63 (0.44–0.79)	0.94 (0.90–0.97)	0.575	0.788 (0.712–0.851)

*n.a.* not applicable due to small numbers

^a^Severe cognitive impairment

^b^Geriatric stroke/neurology, geriatric multi-morbidity

^c^ Orthopedics, urology, geriatric stroke/neurology, geriatric multi-morbidity

#### Interrater reliability

Each assessor in the university hospital (n = 34) conducted 1–42 (Md 4) assessments with 4AT, and the assessors in the county hospital (*n* = 3) 56–75 (Md 69) assessments each. It was possible to calculate interrater reliability with Kappa in 44 of the paired assessments in the university hospital and in 100 in the county hospital (*n* = 144). The strength of agreement between the assessors was 0.918 (*p* < 0.001) overall (n = 144), 0.813 (*p* < 0.001) in the university hospital, and 0.969 (*p* < 0.001) in the county hospital. In the 200 paired assessments with 4AT there was complete agreement in the alertness score, while the attention score (Months backwards) differed in ten paired assessments, AMT4 in six, and acute fluctuation in three.

### Clinical applicability

#### Time duration

The patients and the assessors in the county hospital used 1–7 min (mean 2 min 53 s) to complete the 4AT, and 4 min 30 s - 29 min (mean 12 min 46 s) to complete the OBS scale. The 4AT was completed in ≤2 min for 44% of the patients and ≤ 3 min for 74%. More time was used to complete the 4AT for patients with delirium (reference standard) (*p* = < 0.001), and for patients with dementia (*p* = 0.023) (Mann-Whitney U test). Additional time was also needed by the assessors to conduct a dialog with next of kin or care providers when a patient had memory or communication difficulties, but that time was not measured.

#### Patients’ experiences of being evaluated with the 4AT

Of the 200 patients, 180 (90%) responded to how they experienced being evaluated with the 4AT. The qualitative content analysis of their experiences resulted in the following generic categories: (1) Easy and user-friendly; (2) Associated with difficulties and concerns; and (3) Challenging and demanding.

Of the 180 responding patients, a majority (*n* = 172) experienced the 4AT to be ***easy and user-friendly***. They described the questions as being relevant, interesting, and not unpleasant to answer. Some patients who had expected more difficult questions, and questions requiring pre-knowledge, thought that the 4AT was just a warm-up. Even in the presence of memory problems, the questions were not always experienced as being difficult to answer. A patient commented: *“The questions were not so difficult to answer, it was quite simple, but you are not 25 years anymore … can’t remember”.*

However, a few patients (*n* = 8) experienced the assessment as being *associated with difficulties and concerns.* They described the questions as being tough, boring, a bit strange and difficult to answer*.* For example, they were not used to answering questions about their current location*.* One patient reflected on the benefit of the questions in the 4AT *“It was a little weird sometimes. Can’t imagine that it will be of any use, but I guess it certainly can”.*

Regardless of whether the 4AT and its questions were considered easy and user-friendly or associated with difficulties and concerns, some patients (*n* = 15) experienced the item Months Backwards as the most *challenging and demanding* in the 4AT. Several of these patients said that they had never performed the task before, and some had to stop counting and think instead. One patient described performing this task as follows: *“It was harder than I thought it would be. I will practice months backwards the rest of the week”.*

#### Assessors’ experiences of using the 4AT

The qualitative content analysis of the 14 responding assessors experiences of using the 4AT resulted in the following generic categories: (1) Easy and user-friendly in daily practice; (2) Potential to improve patient care and patient safety; (3) Evoked emotions in the patients; (4) Evaluation challenges, and (5) Doubts about the usefulness and significance of the tool.

The 4 AT was experienced to be easy and user-friendly in daily practice by the majority (*n* = 12) of the responding assessors. It was experienced to be short, uncomplicated, and easy to use by all healthcare professionals without special training. The experience was that most of the patients reacted positively to the test, they participated and tried to respond as best they could without major problems. However, for some patients the assessment was challenging and took longer, especially the Months Backwards test. Assessors thought that the 4AT was a good support tool that rapidly provided a clear picture of the patient’s condition, and that it would be easy to incorporate the 4AT in daily practice and apply it to different care situations. The usefulness of the 4AT was expressed by one assessor as “*Absolutely useful as a routine test, and as a ‘must test’ for old people”.*

According to the assessors who experienced the 4 AT as easy and user-friendly (n = 12), the use of the 4AT was experienced as affording the potential to improve patient care and patient safety. The possibility of early detection of delirium was described as enhancing and facilitating the provision of appropriate treatment and support: *“Assessment tools are an important part of data collection that can provide better care and care planning for older patients”.* Predetermined questions, clear scoring, and the fact that cognitive screening was incorporated in the 4AT were seen as advantages by the assessors. Another described advantage was that the 4AT provided standardized mutual language and concepts, which provided a more objective assessment than subjective interpretations that the assessors believed to be associated with a risk of undetected delirium: “*When the same assessment tool is used by everyone in the personnel group and for all patients, the risk of misjudgment is reduced”.* In addition, assessors were of the opinion that using the 4AT could increase the awareness of delirium among professionals.

When the patients had difficulties responding, assessors reported that the tool was experienced to evoke various emotions in the patients (*n* = 9), such as irritation, frustration, anxiety, and anger. In some cases, when a patient was unable to answer a question, the assessors interpreted it as the patients feeling undervalued, stupid and offended. Patients suffering from dementia or confusion were identified as showing the strongest emotional reactions. These reactions were thought to affect the assessments: “*Sometimes it* (the assessment) *has been difficult and sometimes OK. It depends on how the patient reacts, because usually the patients become angry and describe the questions as ridiculous”.* The importance of being able to adapt to the patient’s condition and reactions and to act *“in the right way”* was highlighted.

Evaluation challenges were described in the use of the 4AT (*n* = 10). Assessors explained that some challenges could be caused by the fact that they were unfamiliar with the 4AT, e.g., that it was difficult to ask the questions in ways that the patient could understand and give a response. The item acute changes was sometimes difficult for the patients to respond to, and there was not always a relative to ask. Assessors had observed that the most demanding and troublesome item for the patients was Months Backwards *“Some items, for example, the Months Backwards from December, was a little tricky for many of the patients*”. The assessors experienced that the Months Backwards item took the longest time in the 4AT and that it evoked emotional reactions. The most challenging evaluation when using the 4AT was to assess patients with severe dementia, severe confusion or language difficulties, which one physician described as *“There were some non-Swedish patients who had difficulties understanding, partly due to cognitive impairment and partly due to language deficiencies”.*

Some assessors (*n* = 5) expressed doubts about the usefulness and significance of the tool. They felt uncomfortable when they conducted the assessment. One assessor experienced the assessment as an interrogation. For another assessor (RN), it was unclear how the score of the 4AT was linked to nursing and nursing actions, but the assessor still thought that the 4AT could be useful for physicians: *“As a nurse, I have not yet seen the connection to nursing and how different scores would lead to different nursing actions”.* Another assessor experienced that the 4AT could not warn of delirium and that the use of standardized screening tools is unnecessary if the professionals have good clinical skills. The assessor thought that it might be important for professionals with less clinical experience to use the 4AT and for professionals who do not trust their own clinical skills. Regarding the use of the 4AT in clinical practice, the importance of information and education about the test was highlighted: *“Of course, it is important to inform and educate about the 4AT, and to include all new colleagues”.*

## Discussion

Our study showed that the Swedish version of the 4AT is an accurate and applicable tool to use in clinical practice for detecting delirium in hospitalized patients across different medical specialties. With a cutoff ≥4, the 4AT showed good overall diagnostic performance, with 88% overall agreement (OPA) in the total sample, and a higher percentage in the general wards (91%). All estimates of accuracy in the general wards were in line with Shenkin et al. [33], who included patients from Emergency rooms and acute geriatric wards.

The AUROC, which helps to estimate the discriminative power of a test, was 0.808 (95% CI = 0.746–0.861) in the total sample, which is considered as very good [51]. In the subgroup patients with dementia, the 4AT was more sensitive, and less specific, in line with Bellelli et al. [32]. This could be expected, as it is known that symptoms displayed by patients with dementia, especially severe dementia [54], may challenge the identification of delirium due to significant clinical overlap [54–56]. It is also known that measures of diagnostic accuracy are sensitive to the characteristics of the population, such as the disease prevalence, the spectrum of the disease, and on the presence of concomitant health problems [51]. In this study, this is reflected by the false positive (*n* = 13) and false negative (*n* = 10) outcomes on the 4AT. Of those 23 patients, 10 hade dementia and 9 had mild cognitive impairment. Additionally, for patients admitted to the special ward for severe cognitive impairment the tool had lower diagnostic accuracy than for patients in the general wards, which might be explained by the fact that most of the patients had moderate to severe dementia, delirium, disorientation, or memory problems in the special ward. Nevertheless, the AUROC was 0.759 (95% CI = 0.635–0.858) in the special ward for severe cognitive impairment, which is considered to be good [51]. As some time passed between the assessments with the 4AT and the OBS scale (median 13 min 30 s), the fluctuating course of delirium may have led to patients displaying symptoms of delirium in one test but not in the other. However, it might be valuable to evaluate the 4AT together with additional measures e.g. inattention [54, 56, 57] in patients with severe cognitive impairment, as delirium can be superimposed on dementia (DSD) [54–57]. In addition, to evaluate 4 AT assessments carried out by clinical professionals when implementing the tool in clinical practice, as in the university hospital in this study, could be valuable.

The total prevalence of delirium with the reference standard was 18% but varied between the general wards (13%) and the special ward for severe cognitive impairment (28%). This was expected due to the differences between the patients’ cause of admission, and the fact that the prevalence of delirium differs by different diagnoses and type of care [14] and, consequently, varies between different hospital wards [21, 58]. The prevalence in the general wards in this study was in line with studies that have reported a prevalence of 12–15% in older patients from a variety of medical specialties [5, 32, 33]. The choice of reference standard probably had less impact on the diagnostic accuracy of our study. Of the few delirium assessments tools available in Swedish, the OBS scale was considered the most appropriate with the lowest degree of subjective interpretation. Additionally, the scale has been used to diagnose delirium in a number of Swedish studies [6, 28, 48, 59]. The analyses of the subscales in the 4AT and the subscales in the OBS scale confirmed the expected correlations between the subscales, which strengthens their concurrent validity. However, surprisingly, alertness in the 4AT showed the lowest correlation with the OBS scale. It is known that alertness is highly specific to delirium, and a very valuable sign clinically [32], but in our study, 198 of the 200 patients scored 0 (normal alertness, not agitated, to mild sleepiness) on alertness, which may have affected the overall diagnostic accuracy negatively. The fluctuating course of delirium may have contributed to the scoring outcome. Another contributing factor could be that the assessment of alertness in the 4AT involves a degree of subjectivity, and that the binary scoring (0 or 4) tends to result in a lower score than a more detailed reference standard assessment [33], such as the OBS scale. Since the cutoff for delirium in the 4AT is ≥4, it might hamper the assessor from using the score of 4 (abnormal) if the patient only displays mild abnormality, e.g., of agitation. Additionally, lack of experience of assessing alertness or insufficient information from reliable informants might result in misjudgment of the patient’s alertness [60].

The item Months backward involves no subjective interpretation. Nevertheless, it was the item that was the most problematic. Ten of 200 paired assessments differed in scoring outcome, and several patients and assessors perceived this item to be the most difficult for the patients. According to the assessors, some patients—above all those with dementia or delirium—became irritated and frustrated when they had difficulties answering the item. These emotional reactions may have affected the assessors’ own attention negatively, with uncertainty about how to handle the patient and the assessment. It is obvious that special attention, knowledge, communication skills, and being able to adapt to the patient’s condition and reactions are required. However, in the Months backwards test it is not consistently defined how to respond to a patient who is struggling with the test, nor is the number of attempts allowed for best performance stated [42]. Not surprisingly, our study showed that it took longer to complete the total 4AT for participants with delirium and/or dementia. According to the assessors, that was most obvious in the Months Backwards test. It has previously been shown that persons with impaired cognitive function [25, 43, 61], above all delirium [25, 62] have difficulties with the performance, especially the backward speed. On the contrary, persons with intact cognitive function can usually complete the task in 15–20 s [42, 43, 61] up to 60–90 s [42]. Cutoff times for the performance have been suggested [42, 43], but are not included in the 4AT. To protect patient comfort and dignity and reduce struggling and frustration, it may be appropriate to define the number of attempts allowed in the 4AT, in combination with time limits in line with the suggestions by Meagher et al. [42]. This may reduce the simplicity of the tool but likely facilitate the assessment situation.

Despite the difficulties described, the strength of agreement in the paired assessments of the 4AT was > 0.8 in the two hospitals. This is demonstrating that the 4AT is reliable for use by different professionals and levels of seniority, as measured in the university hospital. Most patients, and most of the responding assessors, perceived no problems with the 4AT assessment. Assessors believed that the tool could easily be incorporated in daily routines and that it might improve patient outcomes. However, a few assessors expressed doubts about the tool’s usefulness in clinical practice. One assessor’s opinion reflected an over-reliance on his/her own subjective clinical judgment, which has been identified as a cause of unidentified cases of delirium in hospitals [23, 63]. The doubts may reflect a lack of familiarity with using assessment tools, especially for cognitive impairment as in delirium. Even if the 4AT is easy to use by any professional, basic knowledge about delirium and delirium care is needed for an understanding of the benefits of the assessment. The results are similar to those of MacLullich et al. [38], who highlighted the need for increased knowledge about delirium in general and the 4AT in particular. Important is an understanding of which actions are appropriate based on the scoring outcome in the 4AT, e.g., collaboration between medical and nursing professionals, and a more detailed assessment of delirium when the 4AT indicates delirium [38].

Some limitations have to be acknowledged. The most appropriate reference standard assessment, considered the gold standard for the delirium diagnosis, is bedside interviews by physicians using the DSM criteria [64]. However, no physician was available for such a time-consuming assessment and the OBS scale has been used extensively in Swedish research [28]. Several patients with severe cognitive impairment were excluded, which may have led to an underestimation of the delirium rates. Another limitation was that dementia and mild cognitive impairment were not assessed bedside, as these conditions might be underreported in patient records [65]. A strength of the study was the evaluation of accuracy as well as applicability using quantitative and qualitative methods, which is seldom conducted when a test is evaluated. The perspectives from statistical analyses, and the patients’ as well as the healthcare professionals’ experiences of using the 4AT gave a deeper understanding of using the tool. In addition, it provided useful and valuable knowledge for the implementing process of the 4AT in clinical practice. For the analysis of interrater reliability, we used a large sample of assessors (*n* = 144). As geriatric patients have a wide range of diseases and conditions, we evaluated the 4AT in different medical specialties, regardless of the patient’s diagnosis or the cause of the delirium. Therefore, patients with diverse medical conditions were included from two hospitals in different geographical regions, from surgical and non-surgical specialties. The inclusion of patients from a special ward for severe cognitive impairment could be a strength but also a limitation, as the sample became rather diverse. Therefore, in the analysis of accuracy, data were analyzed for the total sample and separately for the different wards.

## Conclusion

Our study indicates that the Swedish version of the 4AT possesses diagnostic accuracy to be used as an assessment tool for delirium detection in older hospitalized patients in a variety of medical specialties. Furthermore, the 4AT is an applicable screening tool for use in clinical practice by different professionals and levels of seniority. It is well tolerated by patients, easy to use for healthcare professionals, and only takes a few minutes to perform. However, the assessor must be particularly observant and focused to ensure correct scoring, especially as the 4AT may evoke emotions if a patient struggles with the test. In conclusion, to improve patient care, patient safety, and patient outcomes, we recommend that the 4AT could be incorporated in the daily routine in clinical practice in healthcare settings in Sweden. Moreover, use of the 4AT as a routine delirium assessment test opens possibilities to use the clinical data in international environments. Future research should address predisposing risk factors for delirium for early identification, particularly in vulnerable patients who thus might be targeted for preventative interventions.

## Data Availability

The datasets supporting the conclusions of this article is not available due to the Regional Ethical Review Board that restricted data to the researchers.

## Abbreviations

4AT: 4 A’s test
AMT4: Abbreviated Mental Test-4
AUROC: Area Under the Receiver Operating Characteristic Curve
CAM: Confusion Assessment Method
DSM: Diagnostic and Statistical Manual of Mental Disorders
ED: Emergency Department
ICD-10: International Statistical Classification of Diseases and Related Health Problems - Tenth Revision
OBS scale: Organic Brain Syndrome scale
RNs: Registered Nurses
STARD: Standards for Reporting Diagnostic Accuracy
QL-QU: Lower Quartile – Upper Quartile
